# The expression of Wnt-1 inducible signaling pathway protein-2 in astrocytoma: Correlation between pathological grade and clinical outcome

**DOI:** 10.3892/ol.2014.2663

**Published:** 2014-11-04

**Authors:** GELEI XIAO, ZHI TANG, XIANRUI YUAN, JIAN YUAN, JIE ZHAO, ZHIPING ZHANG, ZHENGWEN HE, JINGPING LIU

**Affiliations:** 1 The Institute of Skull Base Surgery and Neurooncology at Hunan, Xiangya Hospital, Changsha, Hunan 410008, P.R. China; 2 Department of Neurosurgery, Hunan Provincial Tumor Hospital, The Affiliated Tumor Hospital of Xiangya Medical School, Central South University, Changsha, Hunan 410013, P.R. China

**Keywords:** Wnt-1 inducible signaling pathway protein-2, astrocytoma, progression-free survival, overall survival

## Abstract

Wnt-1 inducible signaling pathway protein-2 (WISP-2) is a member of the CCN family, which is critical for the control of cell morphology, motion, adhesion and other processes involved in tumorigenesis. The expression pattern and clinical significance of WISP-2 in astrocytomas remains unclear. In this study, reverse transcription-polymerase chain reaction was performed to systematically investigate the expression of WISP-2 in 47 astrocytoma tissues of different pathological grades and 10 normal brain tissues. The mRNA expression levels of WISP-2 in the astrocytoma tissues were observed to be significantly higher than those in the normal brain tissues. Furthermore, the upregulation of WISP-2 was found to be associated with astrocytomas of higher pathological grades. Subsequently, 154 astrocytoma and 15 normal brain tissues were analyzed using immunohistochemistry and similar results were obtained. Univariate and multivariate survival analyses were used to determine the correlations between WISP-2 expression and overall survival (OS) and progression-free survival (PFS). The results indicated that the expression of WISP-2 was found to negatively correlate with patient PFS and OS. These results demonstrated that the WISP-2 protein is involved in the pathogenesis and progression of human astrocytomas and may serve as a malignant biomarker of this disease.

## Introduction

Astrocytomas, which are the most common primary tumors of the central nervous system, account for ~one-third of the intrinsic neoplasms of the central nervous system (CNS) (1). Despite recent advances in diagnosis and multimodal therapies, including surgery, radiation, chemotherapy and immunotherapy, the treatment of astrocytomas remains a considerable challenge for neurosurgeons as recurrence is common, however, the underlying mechanism of such remains unclear, and a significant improvement in the survival of patients with high-grade astrocytomas has not yet been achieved (1–5). The recurrence and progression of astrocytomas and the subsequent increase in malignancy accounts for the poor prognosis in patients. These processes are mediated by numerous complex pathways and various regulatory molecules, as well as the tumor microenvironment (6–11). Thus, the molecular mechanisms of the progression of astrocytomas and treatment strategies targeting critical components of astrocytomas have stimulated wide interest.

Wnt-1 inducible signaling pathway protein-2 (WISP-2), also known as CCN5, is a 29-kDa secreted protein that is a member of the connective tissue growth factor (CTGF) and nephroblastoma over-expressed gene family of matricellular proteins, and is critical in growth factor mediated cell proliferation (12–14). This family exhibits conserved multi-modular domains with diverse biological functions, including angiogenesis, stem cell differentiation and carcinogenesis (15). WISP-2 has functions towards the promotion and arrest of cell growth, depending on the cell type and the surrounding microenvironment (16,17). For example, the mitogenic action of estrogen-receptor-positive, non-invasive breast tumor cells relies on the overproduction of WISP-2 by epidermal growth factor or insulin-like growth factor (IGF-1) (18,19). Furthermore, WISP-2 also acts as a growth arrest-specific gene in vascular smooth muscle and prostate cancer cells (20). In addition, it is hypothesized that WISP-2 is significant in preventing the progression of pancreatic cancer, as it is involved in morphological alterations during the mesenchymal to epithelial transition of pancreatic adenocarcinoma and breast cancer cells (21,22). However, the clinical significance of the expression of WISP-2 in astrocytomas of different grades has not been previously investigated. Such studies may aid with the identification of novel targets that enable the development of effective prognostic and therapeutic strategies for the treatment of astrocytoma.

The current study determined the levels of WISP-2 expression in astrocytomas and normal brain tissues using reverse transcription (RT)-polymerase chain reaction (PCR) and immunohistochemistry. In addition, the correlation between WISP-2 expression and progression-free survival (PFS), as well as overall survival (OS) was investigated to obtain a greater insight into the importance of WISP-2 in tumor invasion and recurrence.

## Materials and methods

### Tissue samples

A total of 47 fresh tumor samples were surgically resected from treatment-naive astrocytoma patients (25 males and 22 females, mean age, 38.4 years; range, 2–71 years) with consent obtained prior to treatment at the Department of Neurosurgery, Xiangya Hospital (Changsha, China). For comparison, 10 normal brain tissue samples were isolated from patients undergoing internal decompression surgery following brain trauma. None of the 10 patients exhibited any evidence of cancer or mydriasis.

### Tissue slides

A total of 154 astrocytoma and 14 normal brain paraffin-embedded samples were obtained from the Human Brain Glioma Bank and Brain Tissues Bank, Department of Pathology, Xiangya Hospital (Changsha, China). All patients were admitted to Xiangya Hospital and underwent microsurgical treatment between 2002 and 2008. All of the patients’ follow-up data were available and medical records were reviewed to obtain the following data for each patient: Age, gender, tumor size, extent of tumor removal, pathological diagnosis, PFS and OS. The patient characteristics are shown in Table I.

All specimens were collected and handled according to the protocols approved by the Ethics Committee of Xiangya Hospital. In this study, written informed consent signed either by the patients themselves or their guardians was obtained for all patients.

### RT-PCR

Total RNA was isolated from human brain tumors and normal tissue using Trizol reagent (Invitrogen Life Technologies, Carlsbad, CA, USA). The first strand of cDNA was synthesized from 1 μg of total RNA by using an Oligo (dT) 18 primer and M-MuLV RT (Thermo Fisher Scientific, Waltham, MA, USA). The PCR was performed under the following conditions: 95°C for 5 min, 30 cycles of 95°C for 30 sec, 56°C for 30 sec, 72°C for 30 sec and a final extension step at 72°C for 10 min, using the Taq DNA polymerase kit (Takara Biotechnology Co., Ltd., Dalian, China). The primer sequences used were as follows: Forward, 5′-TTTCTGGCCTTGTCTCTTCC-3′ and reverse, 5′-GTGTGTGTAGGCAGGGAGTG-3′, for human WISP-2 cDNA; forward, 5′-GTCAGTGGTGGACCTGACCT-3′ and reverse, 5′-AGGGGAGCTTCAGTGTGGTG-3′ for glyceraldehyde 3-phosphate dehydrogenase (GAPDH). GAPDH was used as an internal control. The lengths of the WISP-2 and GAPDH amplicons were 155 and 400 bp, respectively. Following PCR, the PCR products were electrophoresed in 1.5% agarose gels. Densitometric analysis was carried out using the Gelpro4.0 image analysis software (Media Cybernetics, Inc., Rockville, MD, USA). The expression of WISP-2 was calculated relative to that of GAPDH in the same sample.

### Immunohistochemistry

Sections of 5-μm were cut from formalin-fixed tissues embedded in paraffin blocks and mounted onto polylysine coated slides. The sections were dewaxed in xylene (Sigma-Aldrich, St. Louis, MO, USA), rehydrated in solutions of descending alcohol concentrations and blocked with endogenous peroxidase (3% H_2_O_2_). The antigen retrieval was conducted by microwave heating in 1 mM ethylene diaminetetraacetic acid for 10 min. The sections were incubated overnight at 4°C with the primary rabbit anti-human WISP2 polyclonal antibody (1:100; ab38317; Abcam, Cambridge, UK). Immunoreactive complexes were detected using the streptavidin peroxidase system (Thermo Fisher Scientific) and visualized with 3,3′-diaminobenzidine. All tissues were stained with hematoxylin and eosin to confirm the diagnosis histologically. The sections that were not probed with the primary antibody were considered as negative controls. Staining data were obtained for at least two sections per tissue and reviewed by two independent investigators, blinded to all clinical data.

A semi-quantitative approach was based on the intensity of staining, (0, negative; 1+, weak; and 2+, strong) and the percentage of positively stained malignant cells, (0, 0–4%; 1, 5–24%; 2, 25–49%; 3,50–74%; and 4, 75–100%). The final immunohistochemistry scores were obtained using the following formula: Final immunohistochemistry score = values of intensity × percentage counts.

### Long term patient follow up

The detailed case history and intra-operative observations of all the patients were recorded. Long-term follow-up, with regard to long-term survival and tumor recurrence was conducted from 2002 until 2010. The mean duration of follow up was 31.3±17.4 months, range, 3–60 months.

### Statistical analysis

Statistical analyses were performed using SPSS software version 16.0 (SPSS, Inc., Chicago, IL, USA). Data are presented as the mean ± standard deviation. The differences in the variables between the groups were tested using one-way analysis of variance or Student’s *t*-test when the data were normally distributed. The correlation analysis between the WISP-2 immunohistochemical score and the clinical variables (pathological grade, age and gender) was performed using the Kruskal-Wallis H test. The association between PFS or OS and WISP-2 expression was analyzed using log-rank tests and presented as Kaplan-Meier plots. Furthermore, a multivariate analysis was performed using the Cox proportional hazards regression to determine the prognostic effect of WISP-2 expression and potential clinical variables [age, gender, tumor size, extent of resection and World Health Organization (WHO) grade (23)] on PFS and OS. P<0.05 was considered to indicate a statistically significant difference.

## Results

### Expression of WISP-2 mRNA was found to correlate with astrocytoma grade

**Figure 1:**
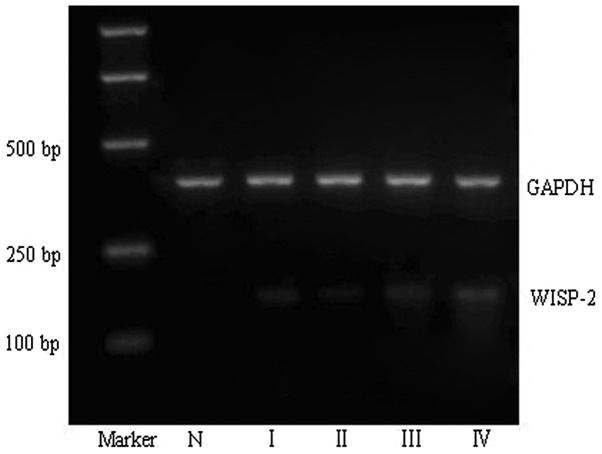
Reverse transcription-polymerase chain reaction analysis of WISP-2 expression levels in normal brain tissues and human astrocytomas of different grades. GAPDH was used as an internal standard. Representative images of electrophoresis of WISP-2 mRNA. A one-way analysis of variance was used for tests of significance: P<0.01, significant difference from the normal control. WISP-2, Wnt-1 inducible signaling pathway protein-2; N, normal brain tissue; I–IV, astrocytoma grades.

The semi-quantitative RT-PCR assay demonstrated that the mRNA levels of WISP-2 in glioma tissues (0.677±0.445) were significantly higher than that in the normal brain tissues (0.172 ± 0.059; P<0.05). Additionally, increased mRNA expression of WISP-2 was found to positively correlate with a higher pathological grade of astrocytoma (P<0.05) (Fig. 1 and Table II).

### Expression of WISP-2 protein correlates with astrocytoma grade

**Figure 2:**
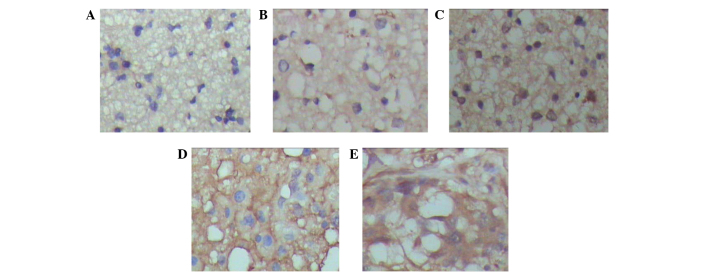
WISP-2 in normal brain and various grades of astrocytic gliomas. (A) Negative WISP-2 expression in normal human brain tissue. (B and C) Weak WISP-2 in stage I and II astrocytomas, respectively, with delicate staining of cytoplasm. (D and E) Markedly increased WISP-2 in malignant stage III and IV astrocytomas, respectively, with positive staining of cytoplasm (magnification, ×400). WISP-2, Wnt-1 inducible signaling pathway protein-2.

The expression of the WISP-2 protein was assessed by immunohistochemistry in paraffin sections in a panel of 154 astrocytomas of various WHO grades and 15 normal brain tissues. In the majority of astrocytomas, diffusive and prominent expression of WISP2 in the cytoplasm of the tumor cells was detected (Fig. 2). The positive expression levels of WISP-2 in the astrocytomas were significantly higher those that in the normal brain tissues. For semi-quantification, WISP-2 expression was significantly associated with pathological grade, however, no association was observed with age, gender or tumor size. The detailed results are shown in Table I.

### Correlation between WISP-2 and patient survival

**Figure 3:**
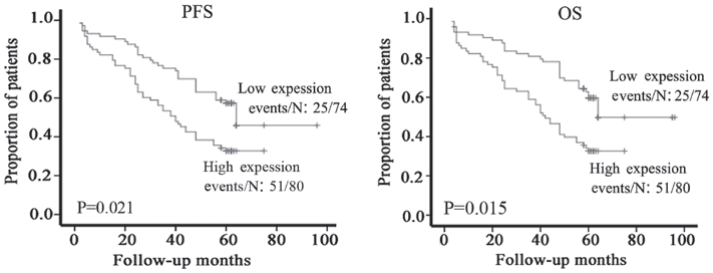
Expression levels of WISP-2 were found to significantly correlate with the survival of astrocytoma patients. Kaplan-Meier estimates of progression-free survival and overall survival are shown according to expression levels of WISP-2 for 154 astrocytomas patients. P-values were obtained using the log-rank test. WISP-2, Wnt-1 inducible signaling pathway protein-2.

The prognostic value of WISP-2 protein expression was also evaluated. Kaplan-Meier analysis was performed to investigate the correlation between WISP-2 expression and patient PFS and OS. The Kaplan-Meier analysis revealed that high WISP-2 expression was significantly associated with a shorter PFS and OS (Fig. 3; P=0.021 and 0.015, respectively). These results indicated that the protein expression levels of WISP-2 may serve as important and independent predictors of survival in astrocytoma patients.

In addition, multivariate Cox’s analysis of the OS and PFS was performed, using WISP-2 expression, tumor size, age, gender and pathological grade as categorical variables. The WISP-2 protein expression level and tumor pathological grade were found to be independent prognostic indicators for patient PFS and OS (P=0.013 and 0.019, Table III, respectively).

## Discussion

Astrocytomas, which are a type of glioma, account for the largest group of primary CNS tumors. The current standard management of glioma is a combined treatment based on microsurgery and including chemotherapy, radiotherapy, immunotherapy and anti-angiogenesis. Malignant astrocytomas are associated with poor prognosis due to their pathological characteristics, including rapid proliferation and diffuse brain invasion (24). To date, a substantial improvement in the treatment of patients with glioma has not been achieved. Therefore, identifying key regulatory molecules in tumor invasion and progression has gained wide interest as they are crucial for the understanding tumor progression and the development of novel interventions (25).

The upregulation of the WISP-2 gene was initially observed in C57MG cells transformed by the Wnt-1 retrovirus (26). Although Wnt family members are critical for numerous developmental processes, and components of the Wnt signaling pathway have been linked to tumorigenesis, the involvement of WISP-2 in mammalian carcinogenesis is not well defined. WISP-2 has been hypothesized to exhibit oncogenic and tumor suppressor activities. The wild type p53 protein has been demonstrated to protect against cancer (27), and the elimination of cells with mutagenic tendencies via apoptosis is critical to its anti-carcinogenic properties. When one or more mutations are present in specific regions of this gene, the tumor suppressor function of the p53 protein is impaired (28). Overexpression of p53 is frequently observed in numerous cancers and is dependant upon the synthesis of mutated forms of the p53 protein. It has also been found to be associated with the malignant progression of various cancers, including procaspase activating compound (29,30). Dhar *et al* (20) demonstrated that the overexpression of oncogenically mutated forms of the p53 gene may be associated with the silencing of WISP-2 during the progression of pancreatic cancer. Furthermore, p53-mutant-induced invasive phenotypes may be mimicked by blocking WISP-2 expression via RNA*i*. Fritah *et al* (31) found that inducing the expression of WISP-2 or supplementing the WISP-2 protein reduces the rate of proliferation, migration and invasion in WISP-2 (−) invasive human breast cancer cells. Previous studies have also shown that the inhibition of miR-10b expression in breast cancer cells induced by WISP-2 is critical for the anti-invasive function of this gene and is mediated via the inhibition of the JNK-HIF-1α-TWIST1 signaling cascades (20,30,32–42). Kouzu *et al* (43) demonstrated that WISP-2 is a reliable independent marker and that downregulation or loss of the WISP-2 gene may be associated with the development of salivary gland tumors.

WISP-2 expression is required for breast tumor cells proliferation in estrogen receptor (ER)-positive human breast cancers. Dhar *et al* (19) reported that IGF-1 induces WISP-2/CCN5 expression via a number of molecular cross-talks and is crucial for the mitogenic switch by the IGF-1 axis in ER-positive breast tumor cells (19). Collectively, the contrasting pathobiological roles of WISP-2, including participation in steroid- and growth factor-induced proliferation and the protection of cells from EMT, migration and invasion, under different microenvironments indicates that WISP2 may be a bifunctional cancer gene and that the major role of WISP-2, under culture conditions, is to protect the cells from adopting invasive phenotypes.

Although several studies have been conducted in different cancer types to elucidate the role of WISP-2 in carcinogenesis and its impact on prognosis, the contribution of WISP-2 in astrocytoma has not been previously investigated.

In the current study, the expression profile of WISP2 was determined at the mRNA and protein levels. WISP-2 expression was found to be significantly upregulated in astrocytoma tissues when compared with normal brain tissues. Furthermore, a significant correlation was identified between WISP-2 protein expression and PFS, as well as OS, which exhibited a highly significant, linear distribution in the Kaplan-Meier analysis (P<0.01). Notably, increased levels of WISP-2 protein expression significantly correlated with a shorter PFS and OS in astrocytoma patients. Although the molecular mechanism requires further study, these results indicate that WISP-2 may be involved in the pathogenesis and progression of astrocytomas. Future studies, using tumor cell lines, such as U251 cells, and WISP-2 gene silencing may be of value with regard to eludicating the association between WISP-2 expression and the proliferation and apoptosis of human astrocytoma cells.

## Figures and Tables

**Table I:** The patient characteristics and the expression of WISP-2 protein in 154 astrocytoma and 15 normal brain tissues.

		WISP-2 (iOD)	
		
Clinical parameter	Patients, n	Mean ± SD	P-value
Tissue type			0.000
Astrocytomas	154	0.986±0.138	
NBT	15	0.157±0.059	
Gender			0.874
Male	80	0.687±0.434	
Female	74	0.666±0.467	
Age (years)			0.618
<40	50	0.642±0.452	
≥40	104	0.708±0.445	
Tumor size (cm)			0.107
<4	89	0.654±0.408	
≥4	65	0.766±0.417	
Pathological grade			0.000
WHO I	23	0.362±0.212	
WHO II	49	0.513±0.347	
WHO III	57	0.769±0.414	
WHO IV	26	0.893±0.473	

iOD, integrated optical density; SD, standard deviation; WISP 2, Wnt-1 inducible signaling pathway protein 2; NBT, normal brain tissue; WHO, World Health Organization.

**Table II:** WISP-2 mRNA expression levels in normal brain and astrocytomas tissues.

		WISP-2 mRNA (iOD)	
		
Clinical parameter	Patients, n	Mean ± SD	P-value
Tissue type			0.000
Astrocytomas	47	0.677±0.445	
NBT	10	0.172±0.059	
Pathological grade			0.000
WHO I	9	0.417±0.276	
WHO II	17	0.634±0.334	
WHO III	15	0.829±0.441	
WHO IV	6	0.941±0.513	

NBT, normal brain tissue; WHO, World Health Organization; WISP 2, Wnt-1 inducible signaling pathway protein 2; SD, standard deviation.

**Table III:** Cox regression analyses of the factors associated with progression-free and overall survival in astrocytoma patients.

	Progression-free survival			Overall survival		
		
Variables	HR	95% CI	P-value	HR	95% CI	P-value
Gender	1.822	0.850–3.906	0.223	1.814	0.841–3.914	0.216
Age	1.000	0.978–1.023	0.990	0.996	0.974–1.019	0.726
WISP-2 expression	1.865	1.225–2.115	0.013	1.971	1.201–3.166	0.019
Tumor size	0.953	0.925–0.985	0.805	1.021	0.901–1.168	0.406
Pathological grade	1.344	0.810–2.231	0.003	1.618	0.863–2.428	0.001

WISP-2, Wnt-1 inducible signaling pathway protein-2; HR, hazard ratio; CI, confidence interval.

## References

[1] Eroes CA, Zausinger S, Kreth FW, Goldbrunner R, Tonn JC (2010). Intramedullary low grade astrocytoma and ependymoma. Surgical results and predicting factors for clinical outcome. Acta Neurochir (Wien).

[2] Wade A, Hayhurst C, Amato-Watkins A, Lammie A, Leach P (2013). Cerebellar pilocytic astrocytoma in adults: a management paradigm for a rare tumour. Acta Neurochir (Wien).

[3] Schuurman PR, Troost D, Verbeeten B, Bosch DA (1997). 5-year survival and clinical prognostic factors in progressive supratentorial diffuse “low-grade” astrocytoma: a retrospective analysis of 46 cases. Acta Neurochir (Wien).

[4] Sturiale CL, Sabatino G, Albanese A (2012). Subcutaneous malignant melanoma of the scalp surgical flap after brain irradiation for anaplastic astrocytoma. J Neurooncol.

[5] Wick W, Platten M, Meisner C (2012). Temozolomide chemotherapy alone versus radiotherapy alone for malignant astrocytoma in the elderly: the NOA-08 randomised, phase 3 trial. Lancet Oncol.

[6] Matsutani T, Nagai Y, Mine S (2009). Akt/protein kinase B overexpression as an accurate prognostic marker in adult diffuse astrocytoma. Acta Neurochir (Wien).

[7] Imaizumi T, Murakami K, Ohta K (2013). MDA5 and ISG56 mediate CXCL10 expression induced by toll-like receptor 4 activation in U373MG human astrocytoma cells. Neurosci Res.

[8] El Hindy N, Bankfalvi A, Herring A (2013). Correlation of aquaporin-1 water channel protein expression with tumor angiogenesis in human astrocytoma. Anticancer Res.

[9] Barton VN, Donson AM, Birks DK (2013). Insulin-like growth factor 2 mRNA binding protein 3 expression is an independent prognostic factor in pediatric pilocytic and pilomyxoid astrocytoma. J Neuropathol Exp Neurol.

[10] Kanoke A, Kanamori M, Kumabe T (2013). Metachronous, multicentric glioma of pilocytic astrocytoma with oligodendroglioma-like component and oligodendroglioma through distinct genetic aberrations. J Neurosurg.

[11] Mascelli S, Raso A, Biassoni R (2012). Analysis of NADP+-dependent isocitrate dehydrogenase-1/2 gene mutations in pediatric brain tumors: report of a secondary anaplastic astrocytoma carrying the IDH1 mutation. J Neurooncol.

[12] Looso M, Michel CS, Konzer A (2012). Spiked-in pulsed in vivo labeling identifies a new member of the CCN family in regenerating newt hearts. J Proteome Res.

[13] Holbourn KP, Acharya KR, Perbal B (2008). The CCN family of proteins: structure-function relationships. Trends Biochem Sci.

[14] Minhas U, Martin TA, Ruge F, Harding KG, Jiang WG (2011). Pattern of expression of CCN family members Cyr61, CTGF and NOV in human acute and chronic wounds. Exp Ther Med.

[15] Jun JI, Lau LF (2011). Taking aim at the extracellular matrix: CCN proteins as emerging therapeutic targets. Nat Rev Drug Discov.

[16] Lake AC, Castellot JJ (2003). CCN5 modulates the antiproliferative effect of heparin and regulates cell motility in vascular smooth muscle cells. Cell Commun Signal.

[17] Schutze N, Noth U, Schneidereit J, Hendrich C, Jakob F (2005). Differential expression of CCN-family members in primary human bone marrow-derived mesenchymal stem cells during osteogenic, chondrogenic and adipogenic differentiation. Cell Commun Signal.

[18] Banerjee S, Sengupta K, Saxena NK, Dhar K, Banerjee SK (2005). Epidermal growth factor induces WISP-2/CCN5 expression in estrogen receptor-alpha-positive breast tumor cells through multiple molecular cross-talks. Mol Cancer Res.

[19] Dhar K, Banerjee S, Dhar G, Sengupta K, Banerjee SK (2007). Insulin-like growth factor-1 (IGF-1) induces WISP-2/CCN5 via multiple molecular cross-talks and is essential for mitogenic switch by IGF-1 axis in estrogen receptor-positive breast tumor cells. Cancer Res.

[20] Dhar G, Banerjee S, Dhar K (2008). Gain of oncogenic function of p53 mutants induces invasive phenotypes in human breast cancer cells by silencing CCN5/WISP-2. Cancer Res.

[21] Dhar G, Mehta S, Banerjee S (2007). Loss of WISP-2/CCN5 signaling in human pancreatic cancer: a potential mechanism for epithelial-mesenchymal-transition. Cancer Lett.

[22] Ouelaa-Benslama R, De Wever O, Hendrix A (2012). Identification of a GαGβγ, AKT and PKCα signalome associated with invasive growth in two genetic models of human breast cancer cell epithelial-to-mesenchymal transition. Int J Oncol.

[23] Louis DN, Ohgaki H, Wiestler OD (2007). The 2007 WHO classification of tumours of the central nervous system. Acta Neuropathol.

[24] Henriksson R, Asklund T, Poulsen HS (2011). Impact of therapy on quality of life, neurocognitive function and their correlates in glioblastoma multiforme: a review. J Neurooncol.

[25] Florian IS, Tomuleasa C, Soritau O (2011). Cancer stem cells and malignant gliomas. From pathophysiology to targeted molecular therapy. J BUON.

[26] Pennica D, Swanson TA, Welsh JW (1998). WISP genes are members of the connective tissue growth factor family that are up-regulated in wnt-1-transformed cells and aberrantly expressed in human colon tumors. Proc Natl Acad Sci USA.

[27] Bargonetti J, Manfredi JJ (2002). Multiple roles of the tumor suppressor p53. Curr Opin Oncol.

[28] Hock AK, Vousden KH (2012). Tumor suppression by p53: fall of the triumvirate?. Cell.

[29] Banerjee S, Dhar G, Haque I (2008). CCN5/WISP-2 expression in breast adenocarcinoma is associated with less frequent progression of the disease and suppresses the invasive phenotypes of tumor cells. Cancer Res.

[30] Banerjee SK, Banerjee S (2012). CCN5/WISP-2: A micromanager of breast cancer progression. J Cell Commun Signal.

[31] Fritah A, Saucier C, De Wever O (2008). Role of WISP-2/CCN5 in the maintenance of a differentiated and noninvasive phenotype in human breast cancer cells. Mol Cell Biol.

[32] Haque I, Banerjee S, Mehta S (2011). Cysteine-rich 61-connective tissue growth factor-nephroblastoma-overexpressed 5 (CCN5)/Wnt-1-induced signaling protein-2 (WISP-2) regulates microRNA-10b via hypoxia-inducible factor-1α-TWIST signaling networks in human breast cancer cells. J Biol Chem.

[33] Chang CH, Fan TC, Yu JC (2014). The prognostic significance of RUNX2 and miR-10a/10b and their inter-relationship in breast cancer. J Transl Med.

[34] Haque I, Banerjee S, De A (2014). CCN5/WISP-2 promotes growth arrest of triple-negative breast cancer cells through accumulation and trafficking of p27 via Skp2 and FOXO3a regulation. Oncogene.

[35] Han X, Yan S, Weijie Z (2014). Critical role of miR-10b in transforming growth factor-β1-induced epithelial-mesenchymal transition in breast cancer. Cancer Gene Ther.

[36] Ibrahim SA, Yip GW, Stock C (2012). Targeting of syndecan-1 by microRNA miR-10b promotes breast cancer cell motility and invasiveness via a Rho-GTPase- and E-cadherin-dependent mechanism. Int J Cancer.

[37] Ferrand N, Stragier E, Redeuilh G, Sabbah M (2012). Glucocorticoids induce CCN5/WISP-2 expression and attenuate invasion in oestrogen receptor-negative human breast cancer cells. Biochem J.

[38] Ma L (2010). Role of miR-10b in breast cancer metastasis. Breast Cancer Res.

[39] Ma L, Teruya-Feldstein J, Weinberg RA (2007). Tumour invasion and metastasis initiated by microRNA-10b in breast cancer. Nature.

[40] Davies SR, Watkins G, Mansel RE, Jiang WG (2007). Differential expression and prognostic implications of the CCN family members WISP-1, WISP-2, and WISP-3 in human breast cancer. Ann Surg Oncol.

[41] Banerjee S, Saxena N, Sengupta K (2003). WISP-2 gene in human breast cancer: estrogen and progesterone inducible expression and regulation of tumor cell proliferation. Neoplasia.

[42] Inadera H, Hashimoto S, Dong HY (2000). WISP-2 as a novel estrogen-responsive gene in human breast cancer cells. Biochem Biophys Res Commun.

[43] Kouzu Y, Uzawa K, Kato M (2006). WISP-2 expression in human salivary gland tumors. Int J Mol Med.

