# The expression of Wnt-1 inducible signaling pathway protein-2 in astrocytoma: Correlation between pathological grade and clinical outcome

**DOI:** 10.3892/ol.2021.12780

**Published:** 2021-05-10

**Authors:** Gelei Xiao, Zhi Tang, Xianrui Yuan, Jian Yuan, Jie Zhao, Zhiping Zhang, Zhengwen He, Jingping Liu

Oncol Lett 9: 235-240, 2015; DOI: 10.3892/ol.2014.2663

Subsequently to the publication of the above paper, an interested reader brought to the readers’ attention that some statements and primer sequences in the paper had apparently been presented incorrectly. The authors have considered the queries raised by the reader, and acknowledge that there were errors featured in the text of the paper and in the two Tables that are in need of correction; therefore, they wish to publish a corrigendum to rectify these issues.

First, the opening sentence of the “*Tissue slides*” subsection of the Materials and methods section, in the left-hand column on p. 236, incorrectly stated that the number of normal brain paraffin-embedded samples was 14; the actual number of normal specimens was 15, in agreement with what was written in Table I. Secondly, in Table I on p. 237, the means of astrocytomas was calculated incorrectly, and the actual mean should have been written as 0.650 ± 0.138 (instead of 0.986±0.138); also, the actual number of astrocytomas of pathological grade WHO I in Table I should have been written as 22, not 23. Thirdly, in Table II on p. 238, the mean of astrocytomas was likewise presently incorrectly owing to a calculation error: This should have been written as 0.694 ± 0.445 (instead of 0.677 ± 0.445).

Finally, the primer sequences for human WISP-2 cDNA were reported incorrectly on p. 236; these should have been written as follows: Forward primer, 5′-TTTCTGGCCTTGTCTCTTCC-3′ and reverse primer, 5′-GCACAGGCGGCGCTGGGTCTC-3′.

Note that these errors do not affect the stated conclusions of the paper. The authors are grateful to the Editor of *Oncology Letters* for allowing them the opportunity to publish this corrigendum, thank the reader of their article for drawing these errors to their attention, and apologize to the readership for any confusion caused.

